# Dalian Institute of Chemical Physics, Chinese Academy of Sciences

**DOI:** 10.1093/nsr/nwz086

**Published:** 2019-09-11

**Figure 1:**
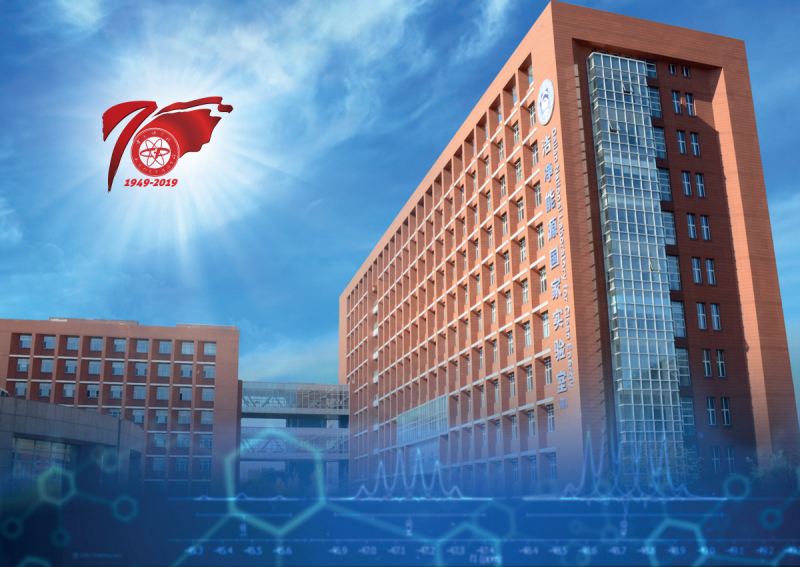


Dalian Institute of Chemical Physics (DICP) is a comprehensive chemical-engineering research institute with a strong international reputation, which has made significant contributions to national economic construction, national security and the progress of science and technology (S&T). With the name of the Scientific Research Institute of Dalian University, the history of DICP began on 19 March 1949. Since then, its name changed several times and the current title of DICP has been adopted since 1970.

Presently, DICP has four campuses in the northeastern China coastal city of Dalian, covering an area of 1.05 million square meters. And the new Energy College is under construction. DICP is host to two state key laboratories, five research laboratories and the Dalian National Laboratory for Clean Energy (drafting), which includes 11 research departments. DICP has more than 200 professors, including 14 academicians of the Chinese Academy of Sciences (CAS) or Chinese Academy of Engineering (CAE), as well as more than 1000 graduate students. These talented researchers are working on diverse basic and application research fields related to chemistry, energy, materials, biotechnology and more. DICP is also collaborating with multiple companies and the Dalian government to further promote technology applications.

In order to support researchers with different ages and levels, DICP established a series of excellent talent support plans in line with national policies, including programs for leading scientists, for young academic leaders, for outstanding young talents and for youth reserve talents.

The achievements of DICP researchers have been recognized by the academia. The technology of ‘Methanol to Olefins (DMTO)’ won the State Technological Invention Awards First Prize in 2014. DICP scientists received a number of seminal prizes in China and abroad: Cunhao Zhang received the Highest National Award of Science and Technology (2013); Xinhe Bao received the Award for Excellence in Natural Gas Conversion (2016) and the Alwin Mittasch Prize (2017); Can Li won the International Catalysis Award (2004) and the Japanese Photochemistry Association Elsevier Lectureship Award (2017); Zhongmin Liu won the AIChE Program Committee's Professional Achievement Award (2018).

**Figure 2:**
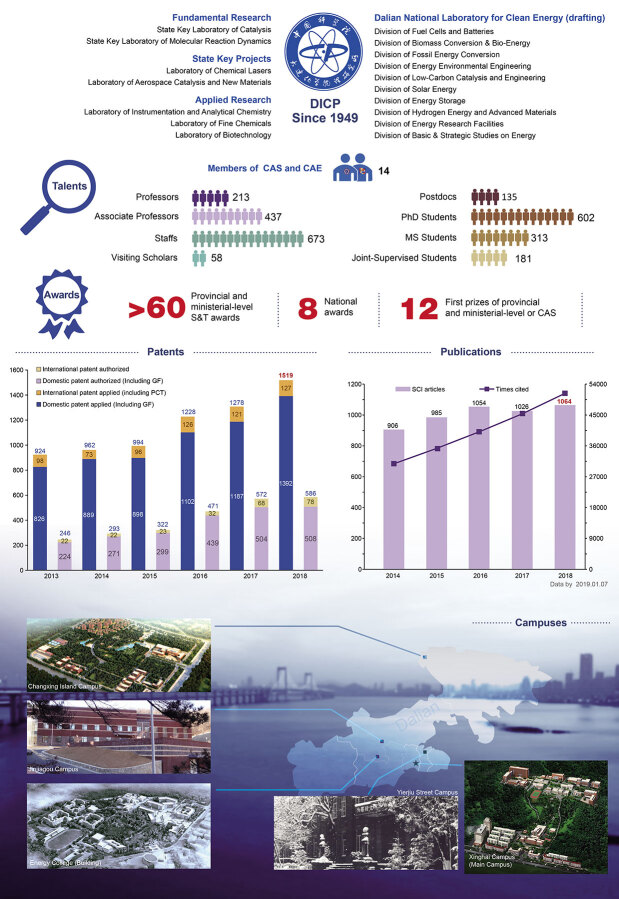


## RESEARCH DIRECTIONS AND ACHIEVEMENTS

### THE BRIGHTEST ULTRAVIOLET LIGHT SOURCE IN THE WORLD

DICP developed the Dalian Coherent Light Source, which is the only free electron laser facility operating in the extreme ultraviolet (EUV) wavelength all over the world. With the laser pulse energy of 210 μJ, it is considered to be the world’s brightest EUV light source.

With the help of the light source and other facilities, the coherent light source team made important progress in many fields. Based on microscopic studies at atomic and molecular scales, the size effect in the structure change of the oxide nanocatalyst has been revealed and the ‘dynamic size effect’ was suggested to illustrate the stability mechanism of the oxide nanostructures. Model systems consisting of 2D overlayers on solid surfaces were applied to study the confinement effect within the 2D nanoreactor and the ‘confinement field effect’ has been suggested to explain the enhanced reactivity of reactions occurring in the confined nanospace.

**Figure 3:**
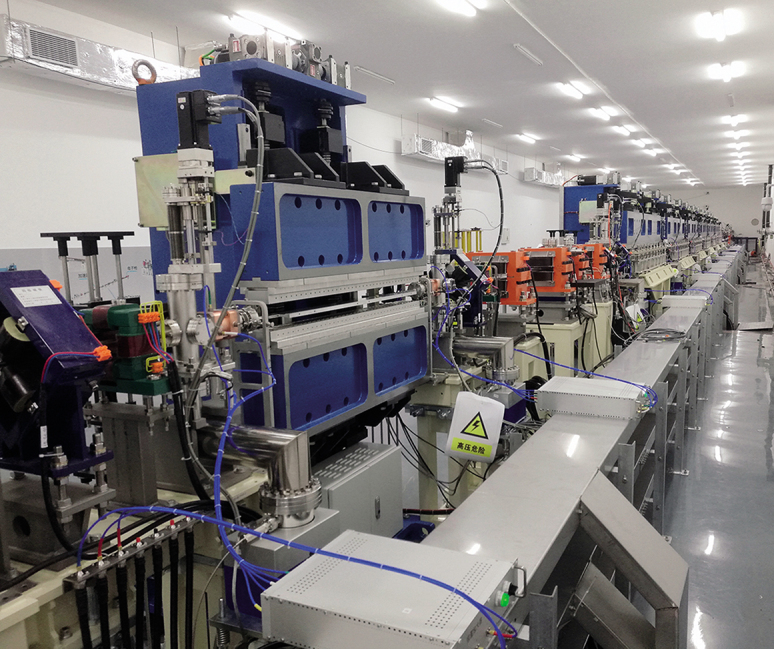
Dalian Coherent Light Source.

## SYNGAS TO ETHANOL AND OTHER FOSSIL-RESOURCE-UTILIZATION TECHNOLOGIES

The world's first demonstration plant for ethanol production via syngas dimethyl ether carbonylation with capacity of 100 000 tons/year has been in operation since 2017. Based on this plant, two commercial construction contracts for future 500 000- and 1 200 000-tons/year plants were signed.

Other fossil-resource-utilization technologies developed by DICP are also changing China's chemical industry, including fluidized bed technology for methanol to propylene, fluidized bed technology for methanol and toluene to para-xylene and olefins, heterogeneous carbonylation of methanol to produce methyl acetate with Ir catalyst, hydrogenation of acetic acid to ethanol, acetic acid and propylene esterification and hydrogenation to produce ethanol and isopropanol, syngas to synthetic oil, ultra-deep desulfurization of diesel and ultra-deep catalytic adsorption desulfurization of gasoline.

**Figure 4:**
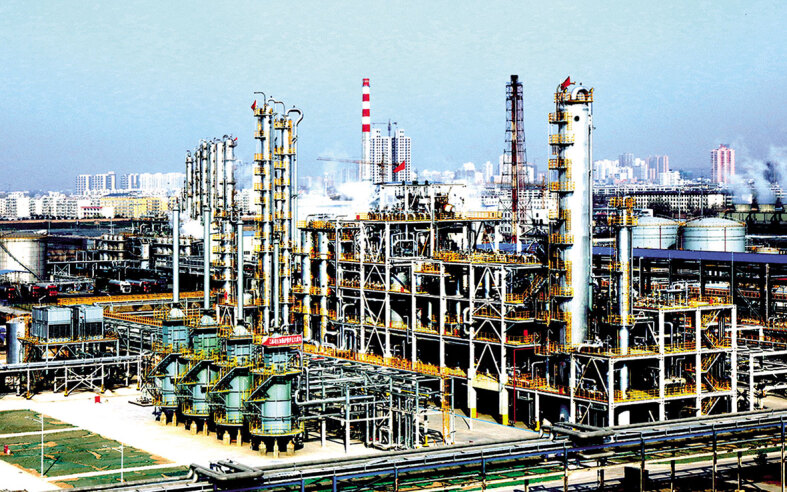
The world's first demonstration plant for ethanol production.

## INNOVATIVE BATTERY TECHNOLOGIES

DICP researchers have put much effort into advanced battery development. For lithium sulfur batteries, they developed and industrially realized high specific-energy lithium sulfur batteries and packs, whose specific energy is world-leading, reaching 609 Wh/kg for the cells and 332 Wh/kg for the packs. The first Chinese large wingspan unmanned aerial vehicle driven by a lithium sulfur battery system was also supported by DICP technologies.

For fuel cells, they designed and put into operation a prototype of a 5-kW high-temperature methanol fuel-cell system and made major contributions to hydrogen fuel cells for vehicles, whose 10 000-unit production line is now under construction.

For liquid-flow batteries, they built a 300-MW/year energy-storage center based on all-vanadium flow batteries and developed the first 5-kWh single zinc-bromine liquid-flow battery demonstration system in China.

**Figure 5:**
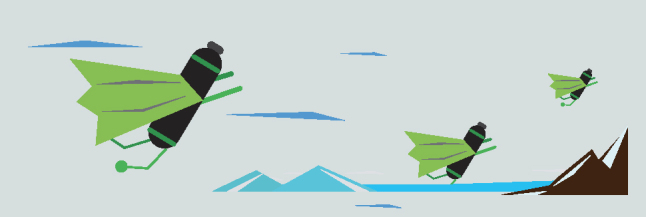

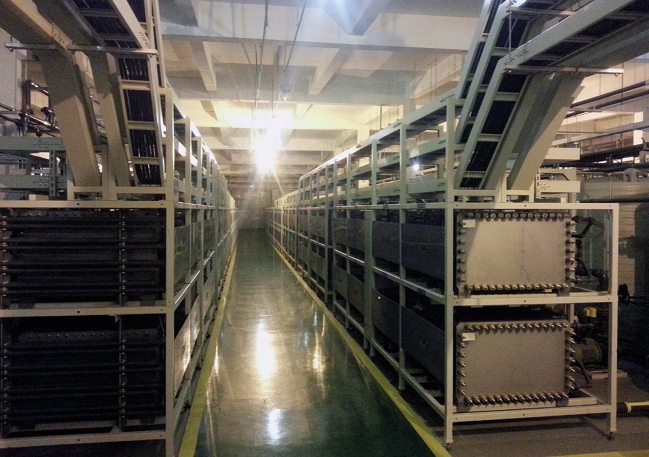
5 MW/10 MWh vanadium flow battery system.

## SOLAR-ENERGY UTILIZATION

DICP has been devoted to the research of catalysis, electrocatalysis and photoelectrocatalysis related to photosynthetic solar fuels production for about 20 years. The team expounded the concept of dual-cocatalysts in photocatalysis, pioneered surface phase junction strategies for charge separation, demonstrated the spatial charge separation between facets in semiconductor photocatalysis, revealed the mechanism of charge separation of semiconductor phase junction in photocatalyst, realized the natural–artificial hybrid system for photocatalytic water splitting, developed a novel mononuclear manganese catalyst with extremely high water oxidation activity comparable to that of natural photosynthesis catalysts and designed a solid solution oxide catalyst for highly efficient CO_2_ hydrogenation to methanol and olefins. The team also independently developed the first instrument in the world for visually observing photogenerated charges in photocatalytic reactions. Based on the breakthroughs in water splitting and CO_2_ hydrogenation catalysts, a pilot demonstration for large-scale solar fuels production has been established, which is the first demonstration using solar energy to synthesize liquid solar fuels in the world.

**Figure 6:**
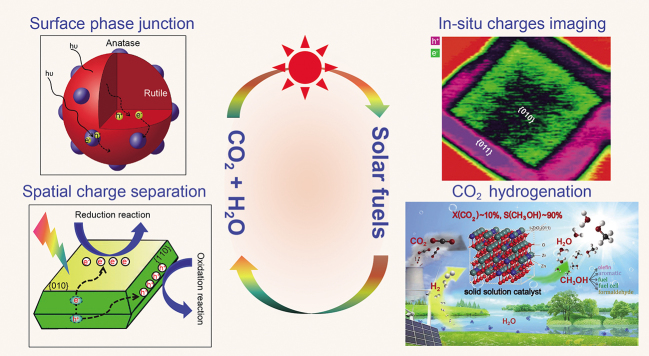
Progresses in artificial photosynthesis for solar fuels production.

## UTILIZATION OF LIGNOCELLULOSIC BIOMASS

Straw, wood chips and other biomass can be used to synthesis-diverse useful chemicals. DICP researchers developed technologies to generate C6 diol and vitamin C-sodium with biomass sugar as the raw material. The process design and production facility construction of 1000-tons/year C6 diol synthesis and 25 000-tons/year vitamin c-sodium synthesis have been finished. The purity of the bio-based C6 diol exceeded 98%.

Methods for the synthesis of aviation kerosene, ethanol, dibasic acid, dibasic alcohol, aromatic hydrocarbons and other chemicals using these biomass raw materials are also being studied in DICP.

**Figure 7:**
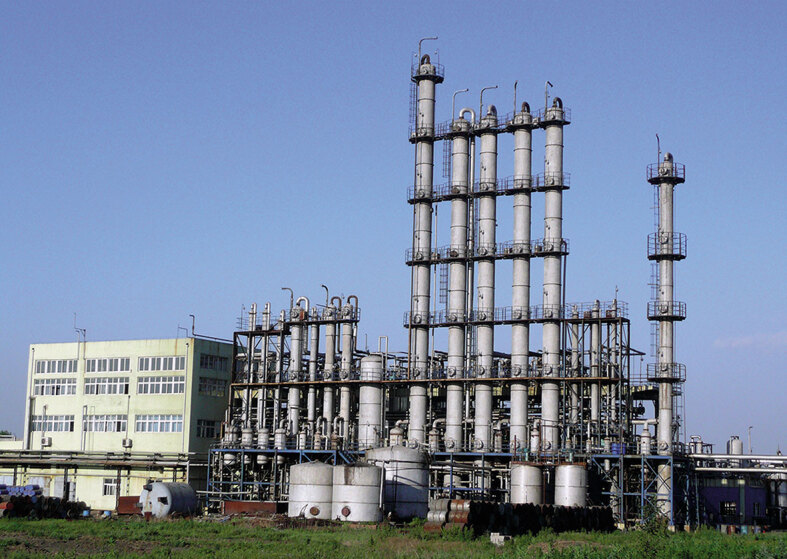
Hydrogenolysis of sorbitol to ethylene glycol and propylene glycol.

## DIRECT CONVERSION OF SYNGAS AND METHANE TO VALUE-ADDED CHEMICALS

Acatalyst design concept of OX-ZEO based on partially reducible metal oxides and zeolites separated CO activation and C-C coupling onto two active sites. It enabled direct syngas conversion to light olefins with a selectivity reaching 80%, surpassing the limit of conventional Fischer-Tropsch synthesis process. Furthermore, it may allow coal-derived syngas with a low H_2_/CO ratio without necessity of the energy- and water-intensive water-gasshift reaction. Exploration of industrial application feasibility of this new technology is under the way.

A catalyst with iron single sites embedded in a silica matrix was developed for direct conversion of methane nonoxidatively to olefins, aromatics and hydrogen. By optimizing the catalyst composition, the loading method and reactor design, methane conversion reached over 30% with the selectivity of ethylene and aromatics over 95%. A single tube test was completed for 1000 hours within the collaborative exploration framework with China National Petroleum Corporation and Saudi Basic Industries Corporation.

**Figure 8:**
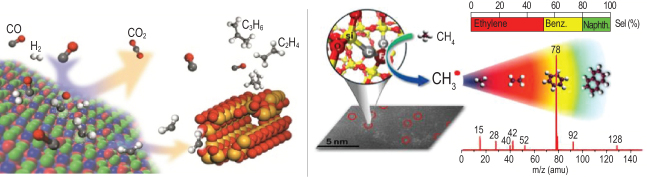
Left: OX-ZEO syngas to light olefins, Copyright 2016, Acta Physico-Chimica Sinica; right: direct methane conversion to ethylene and aromatics.

## MICROREACTION TECHNIQUE

Microreaction processes are chemical processes inside small volumina, typically within microchannels or other sub-millimeter dimensional structures. DICP’s achievements in this field include: a pilot test of isooctyl nitrate synthesis with a capacity of 100 tons/year, with the explosion mechanism revealed and the process safety-control strategy established; a pilot system to continuously nitrify trifluoromethoxy benzene with a throughput of 10 tons/year, with the coupling and control mechanism elucidated and the product quality target of p-nitro side-product content lower than 0.1% achieved; a microreaction system with a capacity of 10 000 tons/year to produce concentrated ammonia solution (>30%), which is expected to be put into production within 2019; the feasibility of chlorination reaction in microreactors to produce food additive and CTP as a rubber additive; a lab-test in cooperation with Karamay Petroleum Company for the regeneration of methyl nitrite, which is one unit in the process of the coal-based production route of ethylene glycol.

**Figure 9:**
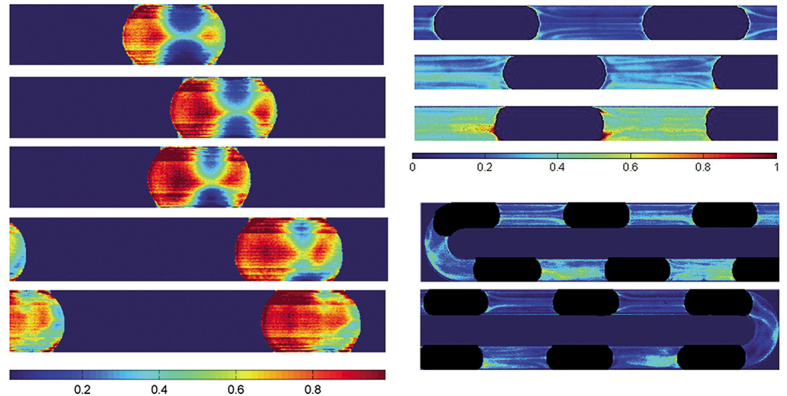
Transport phenomena of multiphase flow in microchannels.

## MULTI-OMICS ANALYSIS IN TRANSLATIONAL MEDICINE

DICP developed deep coverage analysis methods of metabolome, proteome and modified proteome, which greatly improved the coverage of these biotechnologies. A number of original results have been achieved in functional human organ chips and application systems, some of which were among the world’s best.

In the research of metabolic markers for liver cancer, a serum glycolic acid detection-liquid chromatography/tandem mass spectrometry reagent kit developed by DICP was licensed by the Zhejiang branch of the China Food and Drug Administration (Zhexiezhuzhun 20172401284), which is the first licensed mass spectrometry detection kit for hepatobiliary diseases in China. From natural products, DICP researchers found several compounds with target activity for typical chronic diseases and obtained lead compounds with clinical first-line drug activity.

**Figure 10:**
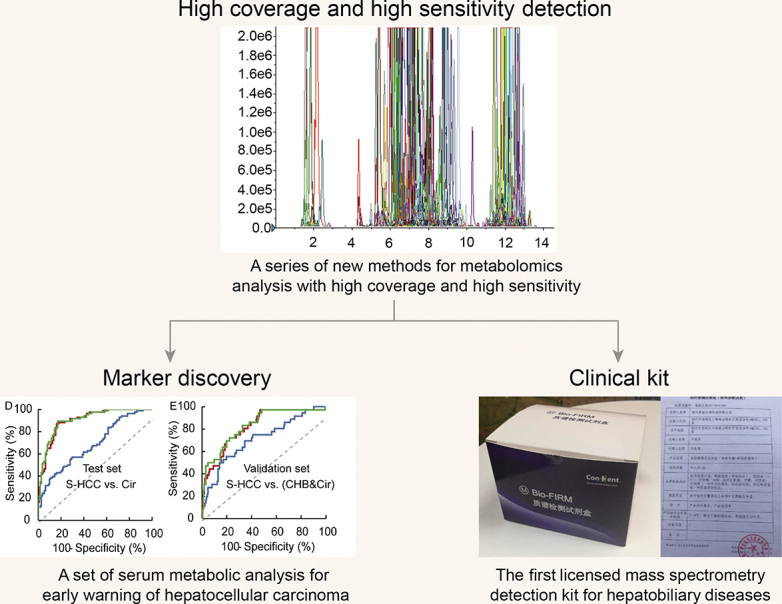


Development and application of high sensitive and high coverage clinical metabolomics technology system.

## OLIGOSACCHARIDE-BASED AGRICULTURAL PREPARATIONS

Oligosaccharide-based agricultural preparations are efficient and environmentally friendly. DICP systematically performed the research, development and application of these novel natural products. The action mechanism of oligosaccharide-induced plant resistance was revealed; eight kinds of highly effective polysaccharide-degrading enzymes were obtained; new biopesticides and biofertilizers were developed; two new large-scale production processes for oligosaccharides were established; a number of standards for oligosaccharides-based agricultural preparations have been drafted.

**Figure 11:**
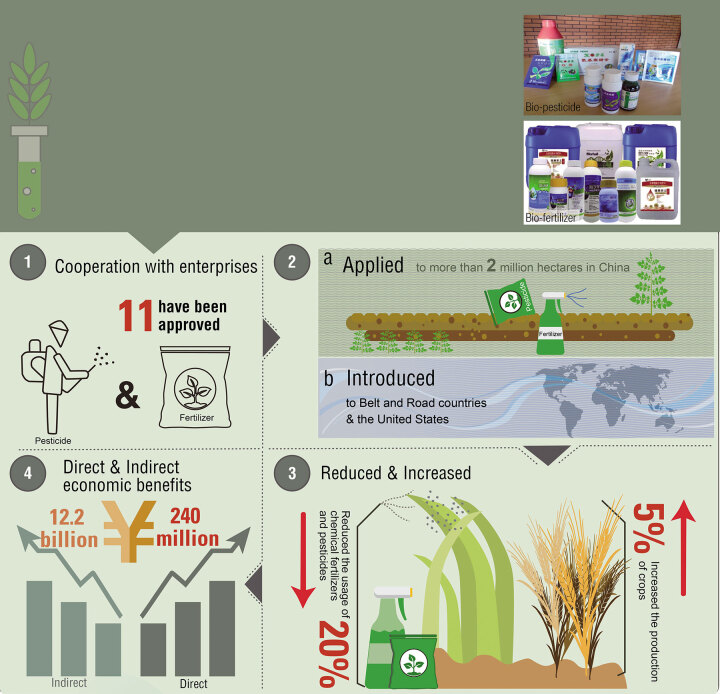


## ENVIRONMENTAL MONITORING TECHNOLOGY AND INSTRUMENT

DICP developed many practical environmental chemical monitoring instruments. The trace harmful gas analytical instrument weighs less than 6 kg and can analyse more than 30 kinds of volatile organic compounds in the closed cabin of a space station. The portable mass spectrometer for roadside identification of drugs can simultaneously identify several illegal drugs in a mixture within 3 seconds at the pg level. Its long-term demonstration application was carried out in Yunnan province, China.

Other instruments developed by DICP include: a vehicle-mounted instrument for the detection of 15 kinds of statutory drugs; rapid-detection equipment for five kinds of common pesticides; a vehicle-mounted time-of-flight mass spectrometer that can analyse 57 photochemical precursors in the atmosphere on urban roads; and an incineration flue-gas and atmospheric dioxin sampler that can perform real-time assessment of flue-gas dioxin toxicity equivalency quantities.

**Figure 12:**
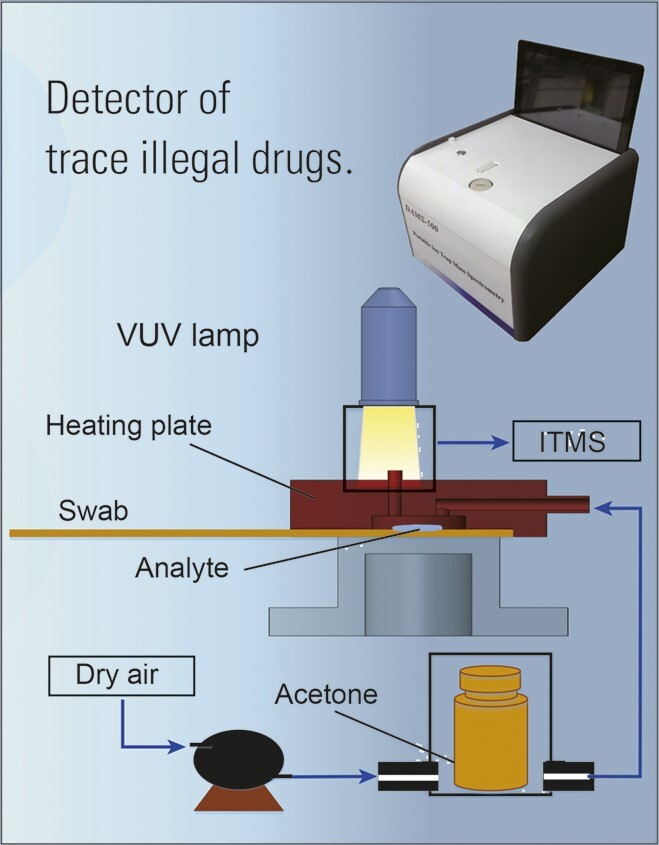


## TRANSFORMATION OF SCIENTIFIC ACHIEVEMENTS

DICP closely cooperates with local governments and enterprises, constantly explored new cooperation channels, and has built an all-factor multi-party S&T cooperation system. DICP built a national demonstration base for entrepreneurship and innovation, a research platform of catalyst amplification and more than 10 achievement transfer bases across the country. These will help to build a bridge between academia and industry, promote the transformation of scientific achievements and serve the local economy.

**Figure 13:**
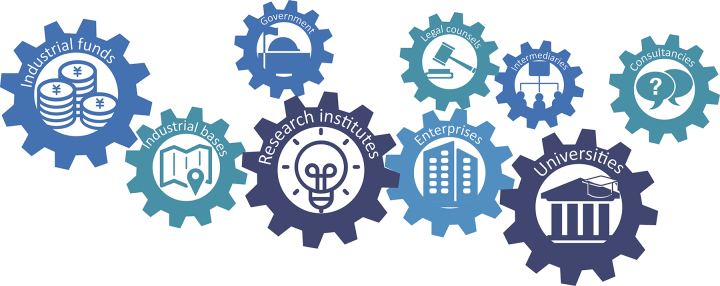
The all-factor multi-party S&T cooperation system

At present, DICP holds 37 companies, covering the fields of the coal chemical industry, catalysis, new energy, membrane separation, environmental protection, instruments, biotechnology, fine chemicals and more, thus forming a high-tech industrial company cluster with relatively complete industrial layout.

In order to provide better financial support for the transformation of scientific achievements, DICP is exploring a new mode that combines scientific research and finance. DICP has set up four types of achievement transfer funds, which will achieve profitability and support scientific research and their commercial applications.

## INTERNATIONAL COOPERATION

There have been nine joint research units established by famous scientific research institutions in France, Britain, the Netherlands and other countries at DICP, including the DICP-BP Energy Innovation Laboratory established in 2007 and the SABIC-DICP Research Center for Advanced Chemicals Production Technology established in 2014.

In 2016, the 16th International Congress on Catalysis was held in Beijing, hosted by DICP. More than 2500 attendees from more than 50 countries attended the conference. In 2017 and 2018, DICP held the International Forum on Clean Energy for two consecutive years. In 2018, more than 300 experts and scholars worldwide attended the meeting.

**Figure 14:**
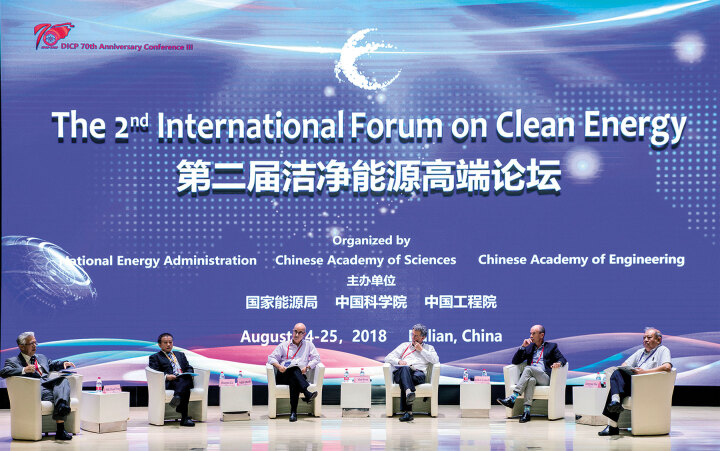
The 2nd International Forum on Clean Energy.

## PUBLIC SCIENCE DAY


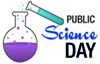


DICP holds the annual Public Science Day, which lasts for two days and is free and open to the public. Public Science Day aims to raise people's interests in science by displaying and promoting scientific knowledge and achievements. By 2019, DICP had successfully held the event for 20 times and received more than 110 000 visitors over the past years. In 2019, the event attracted about 13 000 visitors from all sectors of society, including government agencies, enterprises and institutions, as well as primary, secondary schools and universities, making it the biggest Public Science Day ever held by the institute.

**Figure 15:**
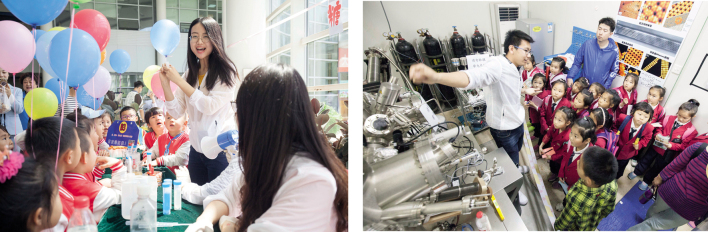


## INTERVIEWS

**Figure 16:**
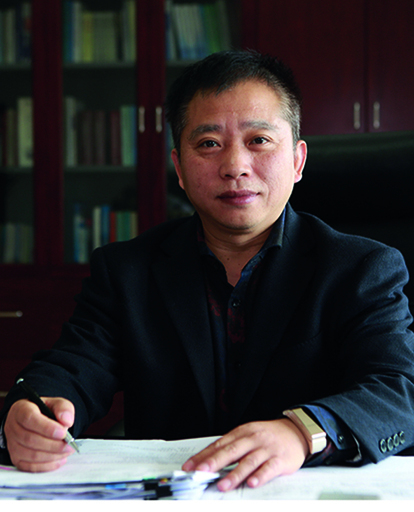
**Zhongmin Liu, Director of DICP** Doctoral degree obtained in 1990; joined DICP in 1990.

**NSR: Please describe DICP with several keywords.**

**Liu:** DICP is committed to ‘innovation, collaboration, rigorousness, and excellence’. I think these words aptly summarize our core values.

**NSR: What are DICP's innovative organization models?**

**Liu:** DICP has designed a ‘group cluster’ program to strengthen collaborations among individual research groups and encourage them to work together towards the institute goals, which include: increasing the competitiveness, strengthening innovative capacity, improving teamwork and meeting national strategic goals. There are two types of group clusters. One is a goal-oriented research center united by loosely connected research groups for a major science and technology project. The other one is a unified research center that is led by one director and consisted of different research groups. The purpose is to promote collaboration and integration across various academic fields and to develop interdisciplinary research directions. In order to cultivate young talents and provide a platform for young researchers to realize their potential, DICP has launched a program entitled ‘Innovative Special-Zone Group’. The group leader is evaluated by DICP Academic Committee after five years. This evaluation process determines whether the group is promoted to formal group or dismissed.

Furthermore, DICP provides a wide range of internal funding opportunities, including matching funds for granted NSFC proposals, supporting funds for outstanding professors, seed money for new ideas, research funds for young researchers, collaborative funds for joint application, and grand challenge funds for major and large-scale problems that can only be addressed by multidisciplinary teams of outstanding researchers. DICP also sets up several technology transfer funds, which are cofinanced with our partners, to accelerate our technology into the market and help our researchers develop industrially relevant ideas as well.

Through the efforts, DICP has established gradually the innovation capabilities to integrate scientific discoveries with the technology development and commercialization.

**NSR: What are your future expectations of DICP?**

**Liu:** A global transition to a clean, efficient and diverse energy system is accelerating. China is calling energy technology revolution for industrial upgrade and sustainable development. As an institute with long history of conducting energy research, DICP puts best efforts to promote the development of energy technology to meet national strategic demands. With establishing the CAS Innovation Center for Clean Energy, DICP has integrated laboratories across CAS institutions to form a strong S&T union in the field of clean energy. Through the efforts of making breakthroughs and demonstrations of transformational and key technologies we will provide cutting-edge theories and technologies to realize the integrated development of fossil energy, renewable energy, and nuclear energy for a clean, low carbon, safe and highly efficient energy system. These approaches will also lay a foundation for the application and establishment of the National Laboratory for Clean Energy. Moving forward, DICP keeps focusing on sustainable energy research and aims to play an indispensable role in national economy and security, and to become a leading research institute in the world.

**Figure 17:**
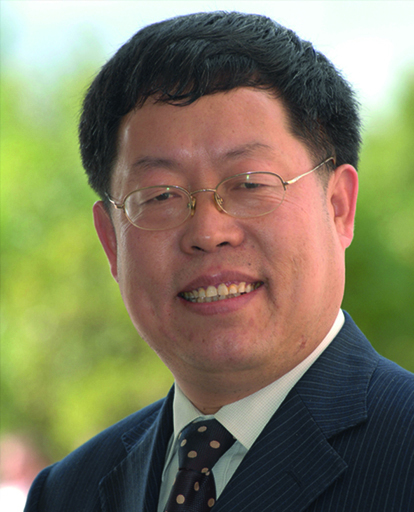
**Can Li** Doctoral degree obtained in 1989; joined DICP in 1989.

**NSR: Please describe DICP with several keywords.**

**Li:** Preciseness, authentic, sureness.

**NSR: What are your research directions? How does DICP support your work?**

**Li:** My research area is catalysis and currently focusing on photocatalysis and electrocatalysis for solar fuels production. I have worked in DICP for almost 30 years. My research group in DICP has been well supported by the institute as well as CAS, the National Natural Science Foundation of China (NSFC) and the Ministry of Science and Technology of China (MOST).

**NSR: You have visited laboratories in many countries. What are the characteristics of DICP compared with other institutes?**

**Li:** The most significant characteristic of DICP is that it features both fundamental and applied researches, which are intimately correlated with each other, especially in catalysis researches.

**Figure 18:**
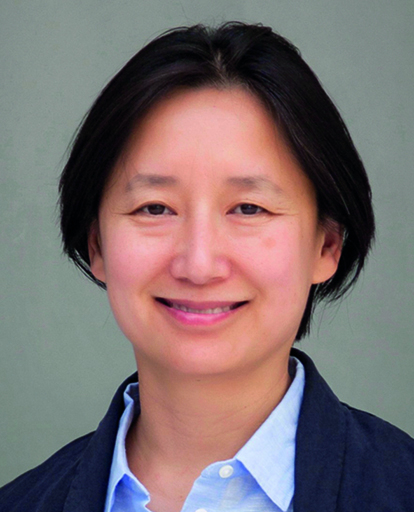
**Ping Chen** Doctoral degree obtained in 1997; joined DICP in 2008.

**NSR: Why did you join DICP?**

**Chen:** DICP has great vision on clean energy and ploughed into the National Laboratory for Clean Energy 10 years ago, which tallies with my research interests and plan. I am pleased to see that such a strategic plan (Clean Energy) has been implemented constantly through the years. As a leading institute in physical chemistry, DICP has a galaxy of distinguished researchers, talented students, advanced research platform and supportive administrative management, which are indispensable to research. The natural beauty and distinct seasons here are bonuses.

**NSR: What are your research directions? How does DICP support your work?**

**Chen:** My research group has been engaged in hydrogen storage and heterogeneous catalysis for hydrogen production and conversion, which are among the key technical challenges in the implementation of hydrogen energy. DICP is building a full chain of hydrogen production-storage-conversion, and thus very supportive to our research via providing a pretty sum of starting-up funding and assisting recruitment of talented researchers.

**NSR: What are the proportions of female researchers and students in DICP? Is DICP friendly for female researchers?**

**Chen:** In academic research, the key to success somehow depends strongly on people's ambition, vision, intelligence and mental/physical strength, etc. Although male researchers are the main stream in DICP, I do see an increase in young female researchers in recent years. I also have a few excellent female students who are among the top students I have had.

**Figure 19:**
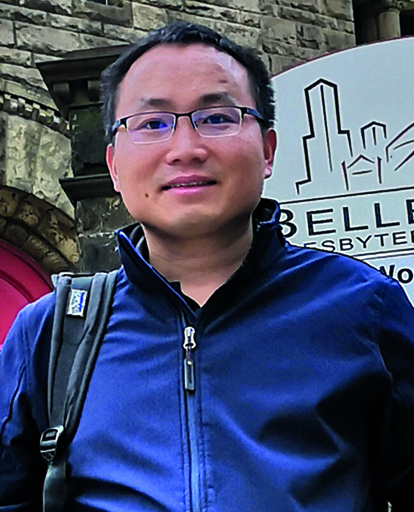
**Dehui Deng** Doctoral degree obtained in 2013; joined DICP in 2013.

**NSR: Why did you join DICP?**

**Deng:** I joined DICP in 2007, as a recommended postgraduate. I was deeply attracted by the academic atmosphere, and encouraged by numerous great achievements of DICP. After my Ph.D. period, I still chose DICP to continue my research career. One of the most important reasons is that the international first-class research platform and the profound cultural deposit of DICP would help me to realize self-worth. Benefiting from the policy of exceptive talents selection of DICP, I was fortunately selected as a ‘100 Talents Program’ researcher and joined as an associate professor upon getting my Ph.D. degree in 2013.

**NSR: How does DICP support young researchers?**

**Deng:** The DICP has a number of opportunities and polices accelerating the growth of young researchers. For young researchers, ‘100 Talents Program’, ‘Excellent Doctoral Talents Program’ and ‘Youth/Excellent Scholar of Zhang Dayu’ programs offer sufficient financial support for the earlier research of those fresh doctoral graduates. In particular, for those outstanding young researchers, they are encouraged to establish their own research groups and will get a start-up funding of at least 5 million Yuan.

**NSR: What are your research directions? How does DICP support your work?**

**Deng:** My research focuses on the modulation of the surfaces and interfaces of 2D materials for the efficient activation and conversion of energy molecules, such as CH_4_, CO, CH_3_OH and H_2_O at mild conditions. I got great support from the exceptive talents selection of ‘100 Talents Program’ by DICP and thus being funded 1 million Yuan, which is the first pot of gold in my research life. I was also supported by the DNL Cooperation Fund and successfully selected as ‘Excellent Scholar of Zhang Dayu’. These fundings and awards greatly promoted the subsequent research running of my team in order.

**Figure 20:**
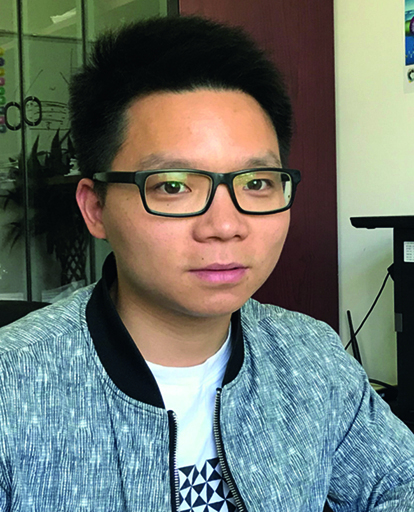
**Kaifeng Wu** Doctoral degree obtained in 2015; joined DICP in 2017.

**NSR: Why did you join DICP?**

**Wu:** My major is physical chemistry. Since DICP is a very prestigious research institute in this area, it is a straightforward and good choice for me to join it. Moreover, the State Key Laboratory of Molecular Reaction Dynamics at DICP, led by famous scientists like Xueming Yang and Donghui Zhang, is a top notch platform for dynamics research around the world. To be able to work with these great scientists is very helpful for my career.

**NSR: How does DICP support young researchers?**

**Wu:** The reason why DICP continues to thrive in the past 70 years is in part because it supports talented researchers of all ages, particularly young researchers. As a young scientist, you can either choose to join a big, well-established group or build your own research group as a principle investigator. In the former case, you can use the best equipment, interact with resourceful colleagues and do cutting-edge research under the guidance of the group leader. In the latter case, DICP gives a very good start-up package to help you build your lab and recruit your crew, with almost 100% freedom, and the group is called a ‘special innovative group’. There is a qualification test for this type of group at the fifth year to determine whether it can become a formal group at DICP, similar to the tenure-track system in the universities in the USA.

**NSR: What are your research directions? How does DICP support your work?**

**Wu:** I work on the time-resolved spectroscopy of optoelectronic materials using ultrafast lasers. In doing so, we aim at elucidating the ultrafast dynamics, such as excited state relaxation and charge and energy transfer, that either contribute to or limit the performance of these optoelectronic materials in various light harvesting and emitting related applications. Using the DICP start-up package, I have now established a ‘special innovative group’. DICP also helps us recruit graduate students and postdoctoral fellows by providing them very good payment. For example, the postdoc salary at DICP is, as far as I know, among the highest in China, which helps me attract some highly qualified Ph.D.s to join the group.

Editors: Yan Zhang and Weijie Zhao; Art editor: Xiaoling Yu.

